# Differential effects of risk factors on the cognitive trajectory of early- and late-onset Alzheimer’s disease

**DOI:** 10.1186/s13195-021-00857-w

**Published:** 2021-06-14

**Authors:** Jaeho Kim, Sook-Young Woo, Seonwoo Kim, Hyemin Jang, Junpyo Kim, Jisun Kim, Sung Hoon Kang, Duk L. Na, Juhee Chin, Liana G. Apostolova, Sang Won Seo, Hee Jin Kim

**Affiliations:** 1 Department of Neurology, Dongtan Sacred Heart Hospital, Hallym University College of Medicine, Hwaseong-si, Gyeonggi-do Republic of Korea; 2 Statistics and Data Center, Samsung Medical Center, Seoul, Republic of Korea; 3 Department of Neurology, Samsung Medical Center, Sungkyunkwan University School of Medicine, Seoul, Republic of Korea; 4 Alzheimer’s Disease Convergence Research Center, Samsung Medical Center, Seoul, Republic of Korea; 5 Department of Neurology, Korea University Guro Hospital, Korea University College of Medicine, Seoul, Republic of Korea; 6 Department of Health Sciences and Technology, SAIHST, Sungkyunkwan University, Seoul, Republic of Korea; 7 Department of Neurology, Indiana University School of Medicine, Indianapolis, IN USA; 8 Department of Intelligent Precision Healthcare Convergence, Sungkyunkwan University, Suwon, Republic of Korea; 9 Department of Digital Health, SAIHST, Sungkyunkwan University, Seoul, Republic of Korea

**Keywords:** Risk factor, Alzheimer’s disease, Age, Cognitive decline

## Abstract

**Background:**

Although few studies have shown that risk factors for Alzheimer’s disease (AD) are associated with cognitive decline in AD, not much is known whether the impact of risk factors differs between early-onset AD (EOAD, symptom onset < 65 years of age) versus late-onset AD (LOAD). Therefore, we evaluated whether the impact of Alzheimer’s disease (AD) risk factors on cognitive trajectories differ in EOAD and LOAD.

**Methods:**

We followed-up 193 EOAD and 476 LOAD patients without known autosomal dominant AD mutation for 32.3 ± 23.2 months. Mixed-effects model analyses were performed to evaluate the effects of *APOE* ε4, low education, hypertension, diabetes, dyslipidemia, and obesity on cognitive trajectories.

**Results:**

*APOE* ε4 carriers showed slower cognitive decline in general cognitive function, language, and memory domains than *APOE* ε4 carriers in EOAD but not in LOAD. Although patients with low education showed slower cognitive decline than patients with high education in both EOAD and LOAD, the effect was stronger in EOAD, specifically in frontal-executive function. Patients with hypertension showed faster cognitive decline than did patients without hypertension in frontal-executive and general cognitive function in LOAD but not in EOAD. Patients with obesity showed slower decline in general cognitive function than non-obese patients in EOAD but not in LOAD.

**Conclusions:**

Known risk factors for AD were associated with slower cognitive decline in EOAD but rapid cognitive decline in LOAD.

## Background

The characteristics of Alzheimer’s disease (AD) differ according to the age of onset in several aspects [1]. Early-onset AD (EOAD) is defined as having an age of onset younger than 65 years old and comprises approximately 5–6% of all AD cases [2]. EOAD patients are reported to show a more atypical (non-amnestic) presentation than do late-onset AD (LOAD) patients, with more hippocampal sparing or posterior cortical atrophy, increased tau burden, and more rapid cognitive decline [3, 4]. Although EOAD differs substantially from LOAD, most AD research is focused on LOAD.

The genetic and environmental risk factors for LOAD have been studied extensively. However, these factors may have different effects on EOAD patients. Apolipoprotein E ε4 (*APOE* ε4), low education, and vascular risk factors are well-known risk factors for AD development [5, 6]. *APOE* ε4 is the strongest genetic risk factor for AD, with an odds ratio of approximately 3 in heterozygotes and 9 to 34 in homozygotes compared to individuals with the ε3/ε3 genotype [3, 7]. Low education also increases the risk of dementia. According to the cognitive reserve theory, less educated individuals do not cope well with pathological burden and have a lower threshold for dementia symptoms [8]. It is also well-established that vascular risk factors such as hypertension, diabetes, dyslipidemia, and obesity are associated with AD [9–13].

Despite the well-established effects of AD risk factors, it is controversial whether these risk factors affect the speed of cognitive decline after symptom onset. Controversy exists about whether the *APOE* polymorphisms are associated with the rate of cognitive decline in AD patients [14–17]. Some studies showed that *APOE* ε4 was associated with more rapid cognitive decline [14, 18, 19] while others showed *APOE* ε4 non-carriers have more rapid cognitive decline [4, 20]. Current evidence indicates that the link between diabetes and the rate of cognitive decline in AD patients is uncertain [21]. Obesity is reported to contribute to cognitive decline by facilitating systemic inflammation [22]. Hypertension is also reported to be associated with cognitive decline in dementia overall [23], but there is limited data in AD specifically. Furthermore, the impact of the aforementioned risk factors on cognitive trajectories according to the age of onset is not well understood, because most longitudinal cohorts consisted of LOAD patients [17, 24, 25].

Therefore, we evaluated the impact of known AD risk factors (*APOE* ε4*,* low education, hypertension, diabetes, dyslipidemia, and obesity) on cognitive trajectories in EOAD and LOAD patients. We tested our hypothesis that the detrimental effect of the risk factors on cognitive decline would be stronger in LOAD patients than in EOAD patients.

## Methods

### Participants

We retrospectively collected 713 AD dementia patients who underwent two or more neuropsychological tests (with at least 1 year interval between each test) and *APOE* genotyping from 2006 to 2013 in the Memory Clinic at the Samsung Medical Center, Seoul, Korea. All patients were of Korean ethnicity. All patients underwent detailed clinical interviews, neurological examinations, neuropsychological tests, and brain MRI at the time of diagnosis. All of the patients met core clinical criteria for probable AD dementia according to the National Institute on Aging-Alzheimer’s Association (NIA-AA) criteria [26]. The patients did not meet other neurodegenerative disease criteria such as those for frontotemporal dementia [27], dementia with Lewy bodies, Parkinson’s disease [28, 29], or subcortical vascular dementia which exhibit severe white matter hyperintensities [30]. We excluded 36 patients who showed stroke or traumatic brain injury that was temporally related to the onset or worsening of cognitive impairment. We also excluded 8 patients who carried causative genetic mutations (*PSEN1*, *PSEN2*, or *APP*). Indications for screening causative mutation were as follows: (1) very early disease onset (< 50 years old), (2) early disease onset (< 60 years old) with two or more affected relatives, or (3) early disease onset (< 60 years old) with one or more affected first-degree relatives with early onset dementia (< 60 years old) [31].

Then, we stratified AD dementia patients according to age of symptom onset based on self-report and/or caregiver-report [32]. The final number of patients included in the analysis was 193 EOAD (onset age < 65 years) and 476 LOAD (onset age ≥ 65 years) patients. The proportion of EOAD (199/669, 28.8%) was larger than known percentage of EOAD among all AD cases (5–6%) [2] since we recruited participants from a referral center. This study was approved by the Institutional Review Board of Samsung Medical Center.

### APOE genotyping

Genomic DNA was extracted from peripheral blood leukocytes using the Wizard Genomic DNA Purification kit following the manufacturer’s instructions (Promega, Madison, WI). Two single nucleotide polymorphisms (SNP; rs429358 for codon 112 and rs7412 for codon 158) in the *APOE* gene were genotyped using TaqMan SNP Genotyping Assays (Applied Biosystems, Foster City, CA) on a 7500 Fast Real-Time PCR System (Applied Biosystems) according to the manufacturer’s instructions.

### Longitudinal follow-up with annual neuropsychological tests

All patients underwent the Seoul Neuropsychological Screening Battery [33, 34] at baseline and one or more times during the follow-up period with at least 1 year interval between each test. The EOAD patients had an average of 3.03 and the LOAD patients had an average of 3.23 longitudinal assessments. Language function was assessed using the Korean version of the Boston Naming Test score (0–60). Visuospatial function was assessed using the Rey Osterrieth Complex Figure Test (RCFT) copy score (0–36). Memory function was assessed by summing the scores of the verbal memory (SVLT recall [0–36], SVLT delayed recall [0–12], and SVLT recognition [0–24]) and visual memory (RCFT immediate recall [0–36], RCFT delayed recall [0–36], and RCFT recognition score [0–12]). Frontal-executive function (0–55) was assessed by summing the scores of the category word generation test (0–20), phonemic word generation test (0–15), and the Stroop color reading test (0–20). General cognition was assessed using the Korean version of the Mini-Mental Status Examination (K-MMSE) (0–30) and the clinical dementia rating sum of boxes (CDR-SB) [35]. These clinical tests were conducted by experienced staffs and supervised by board-certified neuropsychologists.

### Statistical analyses

For the comparison of demographic and clinical data between EOAD and LOAD patients, a two-sample t test and Mann-Whitney test were used for continuous variables, and a chi-square test was used for categorical variables.

For all univariable and multivariable analyses, the linear mixed effect model was used, which was adjusted for random intercept, random slope of time, and baseline age. When we estimated models, the cognitive outcome that did not show a normal distribution was analyzed after natural log transformation. We excluded outliers with an absolute standardized residual > 3. We performed a step-by-step approach to identify the risk factors that have differential effects on the cognitive trajectories of EOAD and LOAD.

First, to evaluate the effect of each risk factor on the rate of cognitive decline in EOAD or LOAD patients, we performed univariable analyses for two-way interactions (risk factor*time). We included age, time, risk factors, and risk factors*time for each risk factor.

Second, to evaluate whether the risk factor affected the rate of cognitive decline in EOAD or LOAD patients when other risk factors were controlled for, we performed multivariable analysis for two-way interactions (risk factors*time). In the multivariable analysis, we included age, time, risk factors, and the interaction between risk factors*time for the risk factors that showed significance in univariable analyses (*P* < .05).

Third, to evaluate whether the risk factors had differential effects on the rate of cognitive decline between EOAD and LOAD patients, we performed univariable analysis for three-way interactions (risk factor*time*group). In the univariable analysis, we included age, time, risk factor, group, two-way interaction effects (risk factor*time, risk factor*group, time*group), and three-way interaction effects (risk factor*time*group) for each risk factor.

Finally, to evaluate whether the risk factors had differential effects on the rate of cognitive decline between EOAD and LOAD patients when other risk factors were controlled, we performed multivariable analysis for three-way interactions (risk factors*time*group). In the final multivariable analyses, we included age, time, risk factors, group, two-way interaction effects (risk factors*times, risk factors*group, time*group), and three-way interaction effects (risk factors*time*group) that showed significance in univariable analyses for three-ways interaction (*P* < .05).

All reported *p* values were two-sided, and a *p* value < .05 was considered to indicate statistical significance. All analyses were performed using SAS software, version 9.4 (SAS Institute, NC, USA) and R version 3.6.1 (R Project for Statistical Computing).

## Results

### Demographics of participants in the longitudinal study

The demographics of participants included in the longitudinal study are described in Table 1. LOAD patients were significantly less educated than the EOAD patients (74.4% vs. 64.8%, *P* < .001). LOAD patients had higher percentage of hypertension and diabetes compared with the EOAD patients (47.9% vs. 28.0%, *P* < .001 and 26.3% vs. 18.7%, *P* = .037, respectively). However, the two groups did not significantly differ in dyslipidemia, sex, *APOE* ε4 carrier proportion, or BMI level.

**Table 1:** Baseline demographics of patients with Alzheimer’s disease by onset age

	All participants	EOAD	LOAD	***p*** value for EOAD vs. LOAD
No.	669	193	476	
Baseline age, years	72.41 ± 8.20	61.97 ± 5.64	76.64 ± 4.41	< 0.001
Onset age, years	69.05 ± 9.25	57.31 ± 5.30	73.81 ± 5.51	< 0.001
Female sex	436 (65.17)	126 (65.28)	310 (65.13)	0.969
Low education (≤ 12 years)*	479 (71.60)	125 (64.77)	354 (74.37)	0.013
*APOE ε4* carrier	341(50.97)	107 (55.44)	234 (49.16)	0.141
Hypertension	282 (42.15)	54 (27.99)	228 (47.90)	< 0.001
Diabetes	161 (24.07)	36 (18.65)	125 (26.26)	0.037
Dyslipidemia	184 (27.50)	52 (26.94)	132 (27.73)	0.836
BMI				
Underweight (< 18.5)	27 (4.04)	6 (3.11)	21 (4.41)	0.002
Normal weight (≤18.5 ~ < 23)	271 (40.51)	85 (44.04)	186 (39.08)	
Over weight (23 ≤ ~ < 25)	182 (27.20)	65 (33.68)	115 (24.16)	
Obesity (≥ 25)	189 (28.25)	37 (19.17)	154 (32.35)	
K-MMSE	20.97 ± 3.96	20.68 ± 3.69	21.08 ± 4.06	0.240
CDR-SB	4.58 ± 2.11	4.38 ± 2.18	4.66 ± 2.08	0.121
Language	33.37 ± 10.71	39.02 ± 10.66	31.07 ± 9.86	< 0.001
Visuospatial	24.07 ± 9.82	21.32 ± 11.20	25.19 ± 8.97	< 0.001
Memory	49.11 ± 11.42	50.96 ± 11.48	48.36 ± 11.32	0.008
Frontal	22.38 ± 9.86	22.60 ± 10.71	22.29 ± 9.50	0.713
Follow-up periods, months	32.38 ± 23.19	31.06 ± 22.31	32.91 ± 23.54	0.352

Continuous variables are expressed as means ± standard deviations

Categorical variables are expressed as frequencies (%)

To compare demographic and clinical data, a two-sample t test was used for continuous variables, and a chi-squared test was used for categorical variables

*12 years of formal education in Korea indicates completion of high school

*APOE ε4* apolipoprotein E e4, *K-MMSE* Korean version of the Mini-Mental State Examination

Overall, baseline cognitive performance was not different between the EOAD and LOAD patients as there was no significant difference in K-MMSE score or CDR-SB score.

Although the follow-up durations varied among participants, it did not differ between EOAD (31.1 ± 22.3 months) and LOAD (33.0 ± 23.5 months) at the group level (*P* = .35).

### The effect of risk factors on cognitive decline in EOAD and LOAD

In EOAD, univariable analyses showed that patients with risk factors demonstrated slower cognitive decline than did those without risk factors. Compared to *APOE* ε4 noncarriers, *APOE* ε4 carriers had slower cognitive decline in general cognitive function (MMSE, and CDR-SB, *P* = .009 and 0.019), language (*P* = .017), memory (*P* = .011), and frontal-executive function (*P* = .001). EOAD patients with dyslipidemia showed slower cognitive decline in memory function (*P* = .011) than did those without dyslipidemia. Patients with hypertension or obesity demonstrated slower cognitive decline in general cognitive function (MMSE, *P* = .002 and CDR-SB, *P* = .021) than did those without these comorbidities. EOAD patients with lower education demonstrated slower cognitive decline in memory (*P* = .029) and frontal-executive function (*P* < .001) than did those with higher levels of education (Table 2).

**Table 2:** Univariable analyses on the effect of risk factors on cognitive decline in EOAD and LOAD

		Language		Visuospatial		Memory		Frontal		MMSE		CDR (sum of boxes)	
		Beta (SE)	*p* value	Beta (SE)	*p* value	Beta (SE)	*p* value	Beta (SE)	*p* value	Beta (SE)	*p* value	Beta (SE)	*p* value
**EOAD**	*APOE ε4* carrier*time	**0.121 (0.050)**	**0.017**	0.041 (0.050)	0.417	**0.151 (0.059)**	**0.011**	**0.154 (0.045)**	**0.001**	**0.064 (0.024)**	**0.009**	− **0.005 (0.002)**	**0.019**
	Low Edu*time	0.079 (0.052)	0.133	0.087 (0.051)	0.088	**0.131 (0.059)**	**0.029**	**0.195 (0.046)**	**< .0001**	0.022 (0.026)	0.405	− 0.000 (0.002)	0.838
	Female*time	0.002 (0.053)	0.967	− 0.031 (0.050)	0.537	0.048 (0.060)	0.422	0.004 (0.048)	0.929	0.013 (0.026)	0.607	0.001 (0.002)	0.597
	Hypertension*time	0.096 (0.056)	0.087	− 0.019 (0.053)	0.717	0.103 (0.061)	0.094	0.095 (0.050)	0.060	**0.084 (0.027)**	**0.002**	− 0.004 (0.002)	0.062
	Diabetes*time	0.060 (0.068)	0.379	− 0.102 (0.063)	0.109	− 0.055 (0.075)	0.463	− 0.066 (0.060)	0.273	0.013 (0.032)	0.680	− 0.003 (0.002)	0.173
	Dyslipidemia*time	0.098 (0.057)	0.086	0.074 (0.054)	0.173	**0.164 (0.063)**	**0.011**	0.062 (0.051)	0.225	0.041 (0.027)	0.138	− 0.002 (0.002)	0.436
	BMI*time (ref:normal weight)												
	Underweight	0.059 (0.141)	0.677	− 0.253 (0.141)	0.075	− 0.043 (0.085)	0.612	3.243 (2.125)	0.128	1.466 (0.884)	0.098	− 0.007 (0.006)	0.264
	Overweight	− 0.022 (0.057)	0.700	− 0.071 (0.055)	0.200	0.035 (0.031)	0.261	− 0.167 (1.069)	0.876	− 0.367 (0.454)	0.420	− 0.001 (0.002)	0.682
	Obesity	0.094 (0.072)	0.190	− 0.089 (0.070)	0.203	0.019 (0.041)	0.635	1.463 (0.984)	0.138	0.215 (0.418)	0.607	− **0.007 (0.003)**	**0.021**
**LOAD**	*APOE ε4* carrier*time	− 0.022 (0.023)	0.345	− 0.026 (0.026)	0.313	− 0.021 (0.029)	0.461	0.011 (0.026)	0.676	0.006 (0.013)	0.648	− 0.002 (0.001)	0.085
	Low Edu*time	0.040 (0.027)	0.132	− 0.055 (0.029)	0.060	0.039 (0.033)	0.230	**0.060 (0.029)**	**0.038**	**0.029 (0.014)**	**0.044**	− 0.001 (0.001)	0.585
	Female*time	− 0.008 (0.025)	0.731	− 0.037 (0.027)	0.175	0.046 (0.030)	0.126	0.046 (0.027)	0.094	− 0.004 (0.013)	0.780	0.000 (0.001)	0.970
	Hypertension*time	0.008 (0.023)	0.723	− 0.009 (0.026)	0.744	− 0.010 (0.029)	0.737	− **0.060 (0.026)**	**0.020**	− **0.030 (0.013)**	**0.021**	0.001 (0.001)	0.278
	Diabetes*time	− 0.037 (0.026)	0.151	− 0.035 (0.029)	0.235	− **0.070 (0.032)**	**0.029**	− **0.064 (0.028)**	**0.026**	− 0.025 (0.014)	0.071	0.001 (0.001)	0.495
	Dyslipidemia*time	0.043 (0.026)	0.098	0.022 (0.029)	0.452	0.030 (0.032)	0.363	0.050 (0.029)	0.082	0.005 (0.014)	0.746	− 0.002 (0.001)	0.144
	BMI*time (ref:normal weight)												
	Underweight	− 0.032 (0.064)	0.618	− 0.060 (0.075)	0.428	0.029 (0.048)	0.549	− **0.192 (0.071)**	**0.007**	− 0.054 (0.035)	0.120	0.001 (0.003)	0.682
	Overweight	0.015 (0.030)	0.624	0.047 (0.034)	0.171	0.007 (0.022)	0.753	0.000 (0.033)	0.989	0.003 (0.017)	0.846	− 0.001 (0.002)	0.500
	Obesity	0.018 (0.028)	0.521	0.039 (0.031)	0.212	− 0.005 (0.020)	0.804	− 0.008 (0.030)	0.785	− 0.010 (0.015)	0.507	0.002 (0.001)	0.242

Univariable linear mixed effects model for each risk factor

Random effect: intercept, time, time*risk factor

In EOAD, multivariable analyses showed that *APOE* ε4 carriers demonstrated slower cognitive decline in general cognitive function (MMSE and CDR-SB, *P* = .011 and .010), language (*P* = .010), memory (*P* = .027), and frontal-executive function (*P* = .002) than did *APOE* ε4 non-carriers. The EOAD patients with higher levels of education demonstrated steeper cognitive decline in frontal-executive function (*P* < .001) than did those with lower levels of education. EOAD patients with dyslipidemia showed slower cognitive decline in memory function (*P* = .044) than did those without dyslipidemia. In addition, patients with hypertension (*P* = .002) or obesity (*P* = .012) demonstrated slower cognitive decline in general cognitive function than did patients without these comorbidities (Table 3).

**Table 3:** Effect of risk factors on cognitive decline in EOAD and LOAD

	EOAD		LOAD			
Cognitive domain	Beta (SE)	*p* value	Beta (SE)	*p* value	*p* value^^^ (risk factor*time*onset age group)	*p* value^^^^, multivariable analysis (risk factor*time*onset age group)
**Language**						
*APOE* ε4 carrier*time	0.130 (0.050)	0.010			0.003	0.005^a$^
**Memory**						
*APOE* ε4 carrier*time	0.131 (0.059)	0.027			0.006	0.011^b$^
High education*time	− 0.094 (0.060)	0.122				
Diabetes*time			− 0.073 (0.032)	0.021	0.083	
Dyslipidemia*time	0.132 (0.065)	0.044			0.030	0.055^b^
**Frontal**						
*APOE* ε4 carrier*time	0.139 (0.044)	0.002			0.005	0.053^c^
High education*time	− 0.169 (0.045)	< .001	− 0.047 (0.029)	0.110	0.017	0.030^c^
Hypertension*time			− 0.065 (0.027)	0.016	0.005	0.016^c$^
Diabetes*time			− 0.062 (0.029)	0.032	0.572	
BMI*time (ref:normal weight)						
Underweight			− 0.197 (0.071)	0.006	0.759	
Overweight			0.015 (0.033)	0.653	0.746	
Obesity			0.023 (0.032)	0.460	0.263	
**MMSE**						
*APOE* ε4 carrier*time	0.063 (0.024)	0.011			0.024	0.026^d^
High education*time			− 0.033 (0.014)	0.017	0.655	
Hypertension*time	0.087 (0.027)	0.002	− 0.022 (0.012)	0.076	< .001	< .001^d$^
**CDR (sum of boxes)**						
*APOE* ε4 carrier*time	− 0.005 (0.002)	0.010			0.230	
BMI*time (ref:normal weight)						0.022^e$^
Underweight	− 0.006 (0.006)	0.304			0.240	0.264
Overweight	− 0.001 (0.002)	0.576			0.959	0.796
Obesity	− 0.007 (0.003)	0.012			0.006	0.008

^^^Univariable linear mixed effects model for each risk factor

Fixed effect: baseline age, onset age group, each risk factor, onset age group*each risk factor

Random effect: time, onset age group*time, each risk factor*time, onset age group*each risk factor*time

^^^^Multivariable linear mixed effects model

^a^Adjusted for: age, education, time, *APOE4*, time*APOE4, group, time*group, group*APOE4, time*group*APOE4

^b^Adjusted for : age, time, group, time*group, *APOE4*, time*APOE4, group*APOE4, time*group*APOE4, education, dyslipidemia, time*dyslipidemia, group*dyslipidemia, time*group*dyslipidemia

^c^Adjusted for : age, time, group, time*group, *APOE4*, time*APOE4, group*APOE4, time*group*APOE4, HTN, time*HTN, group*HTN, time*group*HTN, education, time*education, group*education, time*group*education, sex, group*sex

^d^Adjusted for : age, time, group, time*group, *APOE4*, time*APOE4, group*APOE4, time*group*APOE4, HTN, time*HTN, group*HTN, time*group*HTN, education

^e^Adjusted for : age, time, group, time*group, *APOE4*, time*APOE4, obesity, time*obesity, group*obesity, time*group*obesity

$p < 0.05 after Bonferroni correction for multiple tests (four tests for APOE e4 and two tests for hypertension)

In LOAD, univariable analyses showed that patients with vascular risk factors demonstrated more rapid cognitive decline than did those without vascular risk factors. LOAD patients with diabetes showed more rapid decline in memory function (*P* = .029) and frontal-executive function (*P* = .026) than did those without diabetes. LOAD patients with hypertension showed rapid decline in frontal-executive function (*P* = .020) and general cognitive function (MMSE, *P =* .021). There was no significant impact of *APOE* ε4 carrier status on general cognitive function, memory, or frontal-executive function. LOAD patients with lower education demonstrated slower cognitive decline in frontal-executive (*P* = .038) and general cognitive function (MMSE, *P* = .044). In addition, LOAD patients who were underweight showed rapid cognitive decline in frontal-executive function (*P* = .007) than did those with normal BMI (Table 2).

In LOAD, multivariable analyses, the patients with higher levels of education demonstrated steeper cognitive decline in frontal and general cognitive function (MMSE, *P* = .017) than did those with lower levels of education. LOAD patients with diabetes showed steeper decline in memory function (*P* = .021) and frontal-executive function (*P* = .032) than did those without diabetes. In addition, patients with hypertension showed steeper decline in frontal-executive function (*P* = .016) than did those without hypertension. Underweight patients showed rapid cognitive decline in frontal-executive function (*P* = .006) (Table 3)**.**

### Differential effects of risk factors on cognitive decline between EOAD and LOAD patients

**Fig. 1:**
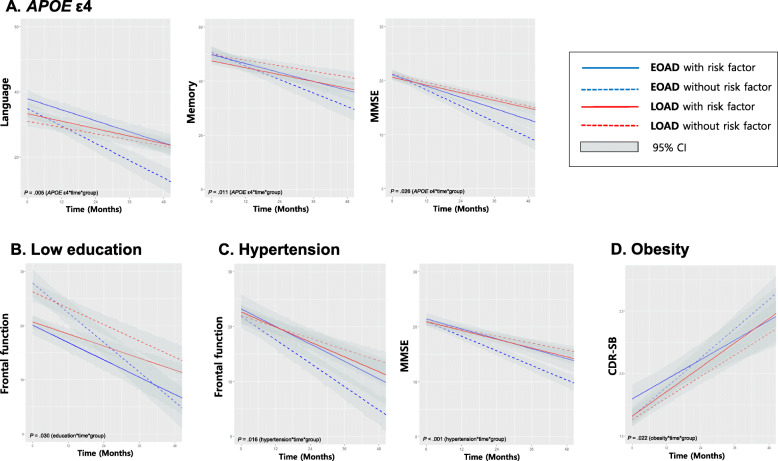
Cognitive trajectories of early-onset Alzheimer’s disease (EOAD) and late-onset AD (LOAD) according to presence of *APOE* ε4 (**A**), low education (**B**), hypertension (**C**), and obesity (**D**)

We evaluated whether the risk factors that were found to have significant effects on the rate of cognitive decline in multivariable analyses in each onset age group (*P* < .05) had differential effects between EOAD and LOAD patients. The results showed that the effects of *APOE* ε4*,* hypertension, low education, and obesity on cognitive trajectories were significantly different in EOAD vs. LOAD patients. *APOE* ε4 carriers showed slower cognitive decline in EOAD but not in LOAD in terms of language (*P* = .005), memory (*P* = .011), and general cognitive function (MMSE, *P* = .026), in which language and memory remained to be significant (*P* < .05) after Bonferroni correction for four multiple tests (Table 3, Fig. 1A). Although low education associated with slower cognitive decline in both EOAD and LOAD patients, the effect was stronger in EOAD patients, and specifically for frontal-executive function (*P* = .030) (Table 3, Fig. 1B). Hypertension associated with faster cognitive decline in LOAD patients but not in EOAD patients in frontal-executive (*P* = .016) and general cognitive function (MMSE, *P* < .001), in which both remained to be significant (*P* < .05) after Bonferroni correction for two multiple tests (Table 3, Fig. 1C). Obesity associated with slower decline general cognitive function in EOAD but not in LOAD patients (CDR-SB, *P* = .022) (Table 3, Fig. 1D).

## Discussion

In this longitudinal study, we evaluated the impact of AD risk factors on cognitive trajectory in EOAD vs. LOAD patients. The major findings were as follows: (1) *APOE* ε4 carriers associated with slower cognitive decline in EOAD but not in LOAD patients. (2) Although low education was associated slower cognitive decline in both EOAD and LOAD patients, the effect was stronger in EOAD patients, specifically for frontal-executive function. (3) Patients with vascular risk factors showed slower cognitive decline in EOAD patients but faster cognitive decline in LOAD patients.

Our first major finding was that in EOAD patients, *APOE* ε4 noncarriers demonstrated a steeper decline in multiple cognitive domains, namely language, memory, frontal-executive and general cognitive function. Meanwhile, in LOAD patients, there was no significant association between *APOE* ε4 status and cognitive decline. According to previous studies, there is controversy about the effect of *APOE* ε4 on rate of cognitive decline in AD. Most published studies used data form the Alzheimer's Disease Neuroimaging Initiative (ADNI) where the cohort consists of predominantly LOAD patients: *APOE* ε4 accelerated hippocampal atrophy in AD, however, there is not enough evidence for the relationship between *APOE* ε4 and cognitive decline [16, 36]. In EOAD, there are studies that show *APOE* ε4 to accelerate [37], decelerate [20], or have no effect [38] on cognitive decline. The controversial results may be due to relatively short follow-up periods and small sample sizes [37–39]. Recent studies further showed that the *APOE* ε4 effect differs according to the cognitive stage [17]. Our current results suggest that the effect of *APOE* ε4 on the cognitive trajectory of language, memory, and MMSE might differ according to the individual’s age of onset. Our result is in line with our previous report showing that in EOAD *APOE* ε4 carriers had less severe brain atrophy in the frontal and perisylvian areas compared to *APOE* ε4 noncarriers while in LOAD *APOE* ε4 carriers showed more severe brain atrophy in the medial temporal area compared to *APOE* ε4 noncarriers [3]. The reason why EOAD showed more rapid cognitive decline in the absence of *APOE* ε4 needs further studies. In attempt to identify genetic risk or cause in these patients, we previously performed whole-exome sequencing in 60 EOAD *APOE* ε4 non-carriers. However, we found only few pathogenic or likely pathogenic variants that may be associated with dementia [40]. There have been several other reports on whole-exome sequencing among a group of patients with EOAD but no novel risk genes were found [41–43]. Collectively, these data suggest that additional and perhaps yet unknown genetic risk factors may be identified by genotyping analyses of larger EOAD cohorts.

Our second major finding was that although patients with higher education demonstrated steeper cognitive decline in both EOAD and LOAD patients, this effect was greater in EOAD patients. The cognitive reserve theory posits that highly educated individuals cope better with AD pathology [8, 44] and do not show dementia symptoms until they have substantial amount of pathological burden in the brain. However, when more educated individuals started to show dementia symptoms, they showed steeper cognitive decline [45]. Likewise, young individuals have greater neural reserve and need greater pathological burden to show dementia symptoms than old individuals [46]. This is supported by pathological studies showing that there were more significant burden of amyloid-ß plaques and neurofibrillary tangles in EOAD compared to LOAD patients [47, 48]. We observed that the impact of cognitive reserve was greater in EOAD compared to LOAD patients, especially in frontal-executive function. This agrees with one previous study showing that AD patients with higher cognitive reserve had better scores on frontal-executive function tests than subjects with lower cognitive reserve [49].

Our third major finding was that EOAD patients without vascular risk factors showed a steeper cognitive decline. EOAD patients without dyslipidemia showed steeper cognitive decline in memory function and EOAD patients without hypertension or obesity demonstrated steeper cognitive decline in general cognitive function. Meanwhile, the reverse association was observed in LOAD patients. LOAD patients with diabetes demonstrated steeper decline in memory function, and LOAD patients with hypertension or diabetes demonstrated steeper decline in frontal function. Diabetes, hypertension, and dyslipidemia are vascular risk factors that are well-known to be associated with the development of AD [6, 9, 10, 21]. However, there is limited published evidence for the association between these vascular risk factor and cognitive decline in AD patients [21]. One previous study showed that vascular risk factors were associated with accelerated brain amyloid-ß accumulation in AD patients [50] via increasing APP expression, reducing clearance of amyloid-ß peptide, inducing oxidative stress, and increasing inflammatory response [51]. However, those studies were generally based on LOAD patients and more studies are needed in EOAD patients. Also, a likely cause for LOAD experiencing more negative effects from vascular risk factors might be that age has reduced overall brain vitality in these subjects, which leads them to be less resilient to the effects of vascular risk factors.

The reason why known risk factors for AD were associated with slower cognitive decline in EOAD patients but rapid cognitive decline in LOAD patients might be explained in several ways. First, it might be related to different pathomechanism of accumulation and clearance of amyloid-ß or tau in EOAD and LOAD [52, 53]. As cognitive decline in dementia stage is more correlated with tau [54], further studies on how the risk factors contribute to tau according to age might be able to provide underlying mechanisms of our results. Second, it is possible that although the pathobiological detrimental effects of *APOE* ε4*,* hypertension, diabetes, and dyslipidemia still exist at younger ages, they might be overshadowed by other yet unknown genetic or environmental factors. Our findings may encourage the search for AD-associated genes in a larger EOAD study sample or in a more homogenous subtype of EOAD, such as young *APOE* ε4 noncarriers without conventional risk factors. Third, the degree of pathological burden might differ according to the presence of risk factors. EOAD patients without risk factor might have needed more amyloid-ß or tau burden to show dementia symptoms [3], which might have led to rapid disease progression thereafter. Lastly, it is possible that the biological effect of vascular risk factors may differ according to age. Vascular risk factors such as hypertension or diabetes might indirectly lead to increased pathological burden in old ages which may result in rapid cognitive decline.

## Strengths and limitations

The strength of our study is that we specifically characterized the impact of *APOE* ε4*,* education, and vascular risk factors in EOAD and LOAD patients in a large longitudinal cohort. To the best of our knowledge, this is the first study to compare the impact of risk factors on cognitive decline in EOAD and LOAD patients.

However, this study also has several limitations. First, AD dementia was diagnosed based on clinical criteria and not confirmed by amyloid or tau biomarkers. Further studies are needed to evaluate whether analysis including amyloid or tau biomarker confirmed AD show similar results. Second, the patients were from a single referral center and our results are not representative of the total AD population. Further multicenter studies including other ethnicities are needed to increase the generalizability of the study results. Third, although we excluded patients when known AD causing mutations (*PSEN1, PSEN2*, or *APP*) were identified, those mutations were not completely ruled out in every AD patient. Lastly, we could not explore the unique pattern of cognitive trajectories of patients who developed AD dementia at oldest old ages due to paucity of data. More studies are needed to explore the genetic or environmental factors that drive rapid cognitive decline in EOAD patients without conventional risk factors.

## Conclusions

In the present study, we evaluated whether the impact of AD risk factors on cognitive trajectories differ in EOAD and LOAD. Our study results suggested that known risk factors for AD were associated with slower cognitive decline in EOAD but rapid cognitive decline in LOAD. More studies are needed to explore the factors that drive rapid cognitive decline in EOAD patients without known risk factors.

## Data Availability

The datasets used and/or analyzed during the current study are available from the corresponding author on reasonable request.

## Abbreviations

AD: Alzheimer’s disease
APOE: Apolipoprotein E
CDR-SB: Clinical dementia rating sum of boxes
CIs: Confidence intervals
EOAD: Early-onset AD
LOAD: Late-onset AD
MMSE: Mini-Mental State Exam
NP: Neuropsychological
PD: Parkinson’s disease
SD: Standard deviations
SMC: Samsung Medical Center
TBI: Traumatic brain injury
